# miRNA-Mediated Fine Regulation of TLR-Induced M1 Polarization

**DOI:** 10.3390/cells13080701

**Published:** 2024-04-18

**Authors:** Noah Rumpel, Georg Riechert, Julia Schumann

**Affiliations:** University Clinic and Outpatient Clinic for Anesthesiology and Operative Intensive Care, University Medicine Halle (Saale), Franzosenweg 1a, 06112 Halle (Saale), Germany

**Keywords:** toll-like receptor, macrophage, miRNA, LPS, LTA

## Abstract

Macrophage polarization to the M1 spectrum is induced by bacterial cell wall components through stimulation of Toll-like family (TLR) receptors. By orchestrating the expression of relevant mediators of the TLR cascade, as well as associated pathways and feedback loops, macrophage polarization is coordinated to ensure an appropriate immune response. This is central to the successful control of pathogens and the maintenance of health. Macrophage polarization is known to be modulated at both the transcriptional and post-transcriptional levels. In recent years, the miRNA-based post-transcriptional regulation of M1 polarization has received increasing attention from the scientific community. Comparative studies have shown that TLR stimulation alters the miRNA profile of macrophages and that macrophages from the M1 or the M2 spectrum differ in terms of miRNAs expressed. Simultaneously, miRNAs are considered critical post-transcriptional regulators of macrophage polarization. In particular, miRNAs are thought to play a regulatory role in the switch between the early proinflammatory response and the resolution phase. In this review, we will discuss the current state of knowledge on the complex interaction of transcriptional and post-transcriptional regulatory mechanisms that ultimately determine the functionality of macrophages.

## 1. Macrophages

Macrophages are innate immune cells that play a critical role in the recognition and clearance of pathogens and cellular debris [1,2,3]. Phenotypic plasticity is a characteristic feature [3,4,5,6,7,8]. Depending on external stimuli, macrophages polarize into distinct functional subtypes [2,4,6,7,8]. The proper regulation of this process is of the utmost importance for the control of pathogens and the maintenance of human health [3,4,6,7,8]. Dysregulation affects both acute and chronic inflammatory responses and is implicated in pathophysiological processes involved in developing and maintaining oncological diseases, autoimmune processes, allergies, obesity, atherosclerosis, and parasitic diseases [3,6,7,8,9]. The functional subdivision according to stimulation is based on the M1-M2 spectrum concept [10]. M1 macrophages are proinflammatory, producing the cytokines interleukin-1beta (IL-1ß), IL-6, IL-12, IL-23, tumor necrosis factor-alpha (TNF-α), and reactive nitrogen and oxygen species [2,6,7,11]. Released cytokines augment inflammation and promote the acquired immune response [2,4]. The reactive nitrogen and oxygen species have direct antibacterial activity but also cause tissue damage as part of the acute pathogenic rejection process [2,4]. In addition, M1 macrophages are characterized by enhanced antigen presentation and antiviral effects [2,4,6]. This makes them critical players in acute inflammation [3,6]. M2 macrophages are antiphlogistic and release anti-inflammatory cytokines such as IL-10 and transforming growth factor-beta (TGF-ß) [2,6,7]. M2 macrophages are also characterized by high phagocytic activity [2,6,7]. They play a key role in promoting wound healing by eliminating apoptotic cells and promoting angiogenesis and fibrosis through released mediators [2,3,4,6,11]. Moreover, M2 macrophages play a pathophysiological role in inadequate immune processes and in tumor progression [3,4,6].

## 2. Macrophage Polarization

### 2.1. Initiation of Macrophage Polarization

Macrophages polarize from naïve monocytes into the M1 or M2 phenotype in response to external stimuli that are recognized by their membrane receptors [8,12]. Signal transduction proceeds via intracellular signaling cascades, which ultimately affect macrophage gene expression. The resulting changes in protein levels lead to functional adaptations and a specialized phenotype [12]. The classical polarization to M1 is induced by bacterial cell wall components (e.g., lipopolysaccharide (LPS), lipoteichoic acid (LTA), lipopeptide, and peptidoglycans) [6,8,11]. An alternative polarization to the M2 phenotype is triggered by colony-stimulating factor 1 (CSF1), TGF-ß, and the interleukins IL-4, IL-10, and IL-13 [6,8,11,13].

Macrophages recognize self and nonself using pattern recognition receptors (PRRs). PRRs bind pathogen-associated molecular patterns (PAMPs) produced by pathogens but not by the body’s own cells. The family of Toll-like receptors (TLRs), which are surface receptors, are among the most immunologically relevant PRRs of macrophages [14,15,16]. TLRs are named after the Toll receptor of the fruit fly *Drosophila melanogaster*, which was discovered in 1985. Initially, it was thought to play a role only in fruit fly embryonic development. In 1996, however, a major role in the defense of the fly against bacteria and fungi was described [15]. Since then, Toll homologs have been identified in insects, plants, and mammals. So far, 13 different mammalian TLR genes have been identified, some recognizing different PAMPs (Table 1) [14,15,16]. Among the most immunologically important Toll-like receptors are TLR4 and TLR2, which are highly expressed by cells of the innate immune response, including macrophages. TLR4 plays an important role in the recognition of Gram-negative bacteria by sensing LPS, a critical component of the Gram-negative bacterial cell wall [14,15,16]. TLR2 recognizes lipoproteins from Gram-negative bacteria as well as LTA from Gram-positive bacteria, with the recognition of Gram-positive bacteria being of particular importance [14,15,16]. All TLRs share an intracellular membrane domain, the TLR/IL-1 receptor domain (TIR domain). This domain is required for interaction with subsequent molecules in the signaling cascade [14,15,16].

**Figure 1:**
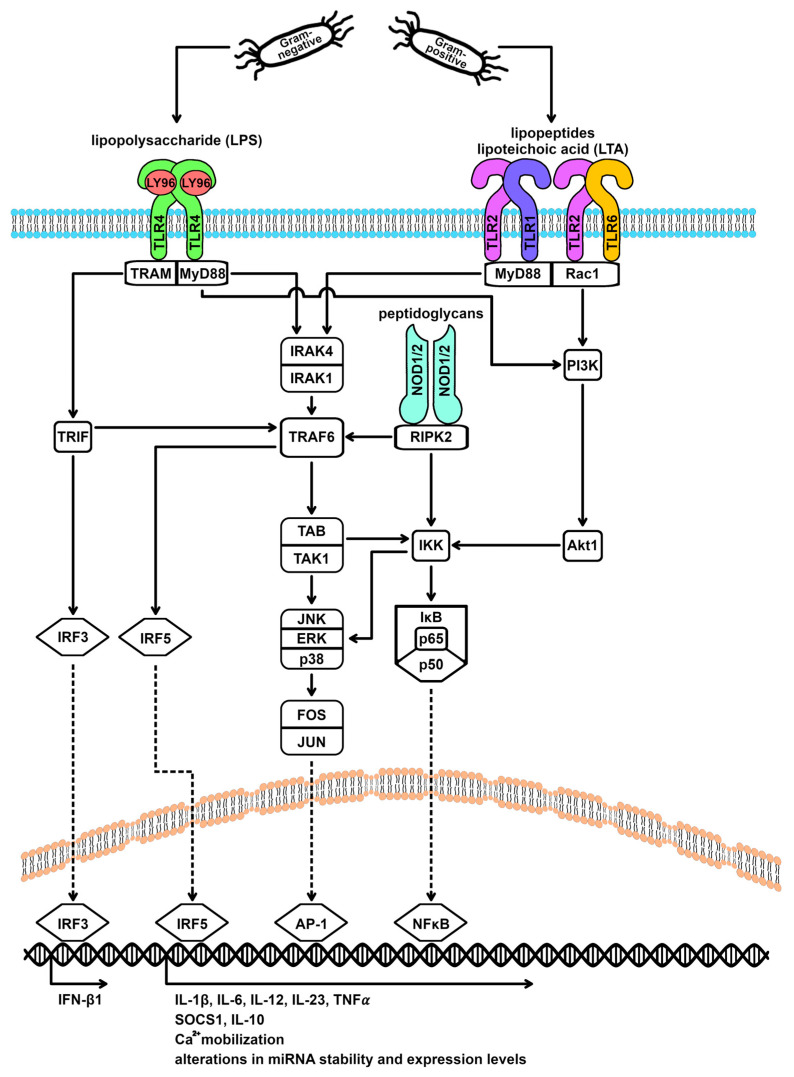
Signaling cascades of TLR4 and TLR2 receptors. Bacteria express molecular structures on their cell surface that are specifically recognized by receptors of the Toll-like receptor (TLR) family. Gram-negative bacteria are characterized by the expression of lipopolysaccharide (LPS), which induces the TLR4 signaling cascade. In contrast, the TLR2 signaling cascade is typically induced by Gram-positive bacteria through the recognition of cell wall components such as lipoteichoic acid (LTA). In the case of Gram-negative stimulation of TLR4, signaling occurs via the adapter proteins MyD88 (MyD88-dependent cascade) and TRIF (MyD88-independent cascade). When Gram-positive bacteria stimulate TLR2, the Rac1-PI3K-Akt signaling pathway is activated in addition to the MyD88-dependent signaling cascade. AP-1 = activating protein-1, Akt1 = AKT serine-threonine protein kinase 1/protein kinase B, ERK = extracellular signal-regulated kinases, FOS = Fos proto-oncogene, IFN-β1 = interferon beta 1, IκB = NFκB inhibitor, IKK = IκB kinase, IL = interleukin, IRAK = interleukin 1 receptor associated kinase, IRF = interferon regulatory factor, JNK = c-Jun N-terminal kinase 1, JUN = Jun proto-oncogene, LY96 = lymphocyte antigen 96, MyD88 = myeloid differentiation primary response 88, NFκB = nuclear factor of kappa light polypeptide gene enhancer in B-cells, NOD = nucleotide binding oligomerization domain, p38 = mitogen-activated protein kinase 14, p50 = NFκB p50 subunit, p65 = NFκB p65 subunit, PI3K = phosphatidylinositol-4,5-bisphosphate 3-kinases, Rac1 = Rac family small GTPase 1, RIPK2 = receptor interacting serine/threonine kinase 2, SOCS1 = suppressor of cytokine signaling 1, TAB = TGF-beta activated kinase 1 binding protein, TAK1 = TGF-beta activated kinase 1, TLR = toll-like receptor, TNF-α = tumor necrosis factor-alpha, TRAF6 = TNF receptor associated factor 6, TRAM = TIR domain containing adaptor molecule 2, and TRIF = TIR domain containing adaptor molecule 1.

Both TLR4 and TLR2 interact with specific coreceptors for ligand binding and initiation of a signaling cascade (Figure 1). TLR4 requires interaction with lymphocyte antigen 96 (Ly96), also known as MD-2, and a cluster of differentiation (CD) 14. The binding of the cell wall component LPS of Gram-negative bacteria is mediated by the membrane protein CD14, which then interacts with the Ly96/TLR4 complex [14,15,16,17]. Two TLR4 receptors then form a homodimer that can interact with the TIR domain-containing adaptor proteins (TIRAMs) MyD88 and TRAM/TRIF [14,16,18,19,20,21,22,23,24,25]. Intracellular signal transduction via the MyD88-dependent cascade involves activation of the kinases IRAK4 and IRAK1, the signal transducer protein TRAF6, the MAP kinase MAP3K7 (also known as TAK1), and finally, the kinases p38, ERK, JNK, and IKK [14,16,18,19,22,23,24,25]. MAP kinase-mediated phosphorylation of the Fos and Jun family proteins affects their stability, DNA-binding activity, and transactivation potential, making them effective as AP-1 transcription factors [14,26,27]. IKK-alpha and IKK-beta, in turn, cause the activation and translocation of the transcription factor NFκB to the nucleus by phosphorylating the IκB inhibitor proteins for degradation [14,15,16,17,18,19,23,24,25]. Furthermore, the MyD88-dependent pathway induces the activation of the transcription factor IRF5 through TRAF6 [2]. TRIF-mediated, MyD88-independent signaling leads to the activation of the transcription factor IRF3 [14,18,19,21,25].

TLR2 forms heterodimers with either TLR1 or TLR6. This heterodimer formation expands the set of possible TLR2 ligands. Together with TLR1, TLR2 recognizes triacyl lipopeptides, and together with TLR6, it recognizes diacyl lipopeptides [14,15,16]. The adapter molecule Rac1 mediates signal transduction by activating the kinases PI3K and Akt1 (Rac1-PI3K-Akt pathway) [12,28]. In addition, the previously described MyD88-dependent pathway can be triggered [14,15,16,17,19,20,22,23,24,25]. Both pathways result in activation of transcription factors AP-1, NFκB, and IRF5 [14,15,19,20,22,23,24,25].

In addition, intracellular recognition of bacterial cell wall components, such as peptidoglycans, by members of the NOD-like receptor family also activates the transcription factors AP-1 and NFκB (Figure 1) [18,29]. AP-1, NFκB, and IRF5 are mediators of M1 polarization and synergistically promote proinflammatory cytokine expression [2,3,4,18,30,31]. IRF5 has also been described as a means of repressing IL-10 synthesis [2].

### 2.2. Regulation of Macrophage Polarization

**Figure 2:**
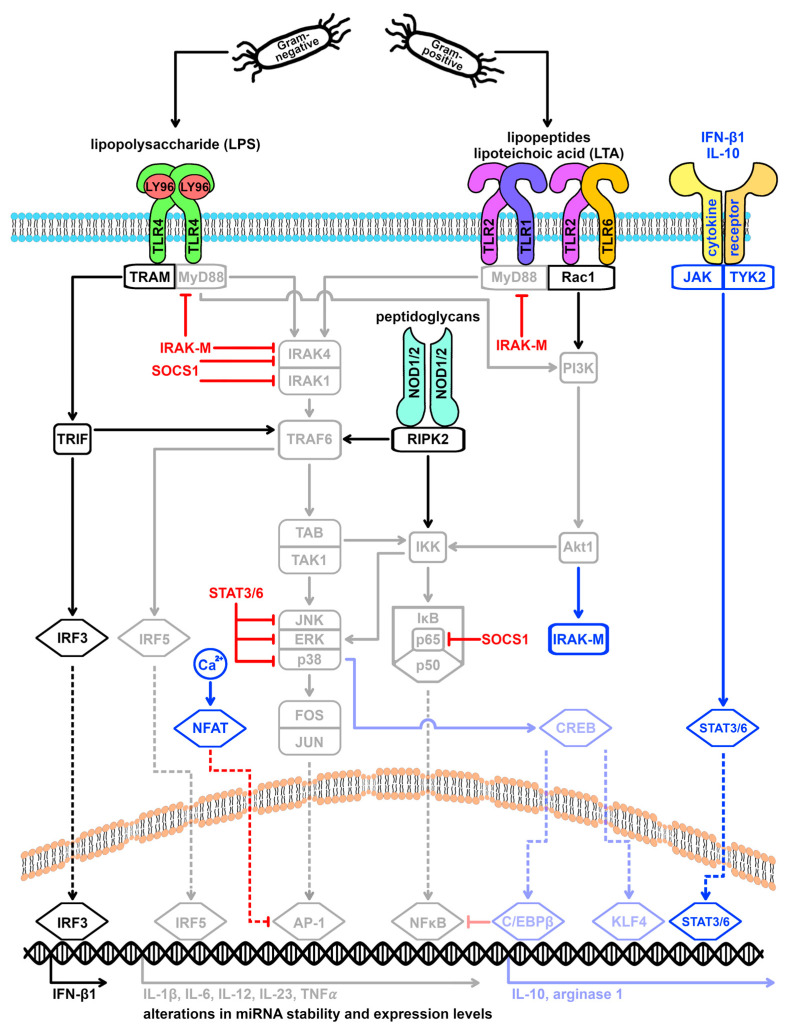
Negative feedback loops within the TLR4 and the TLR2 signaling cascade. Activation of TLR4 by Gram-negative bacteria and of TLR2 by Gram-positive bacteria induces the upregulation of regulatory proteins such as STAT3, STAT6, IRAKM, and SOCS1, as well as transcription factors such as C/EBPbeta, KLF4, and members of the NFAT family. These mediators are known to inhibit central mediators of the TLR signaling cascade and also induce transcriptional expression of M2 spectrum markers. The net effect is an attenuation of macrophage M1 polarization. AP-1 = activating protein-1, Akt1 = AKT serine-threonine protein kinase 1/Protein Kinase B, C/EBPβ = CCAAT enhancer binding protein beta, CREB = cAMP responsive element binding protein, ERK = extracellular signal-regulated kinases, FOS = Fos proto-oncogene, IFN-β1 = interferon beta 1, IκB = NFκB inhibitor, IKK = IκB kinase, IL = interleukin, IRAK = interleukin 1 receptor associated kinase, IRF = interferon regulatory factor, JAK = Janus kinase, JNK = c-Jun N-terminal kinase 1, JUN = Jun proto-oncogene, KLF4 = KLF transcription factor 4, LY96 = lymphocyte antigen 96, MyD88 = myeloid differentiation primary response 88, NFAT = nuclear factor of activated T cells, NFκB = nuclear factor of kappa light polypeptide gene enhancer in B-cells, NOD = nucleotide binding oligomerization domain, p38 = mitogen-activated protein kinase 14, p50 = NFκB p50 subunit, p65 = NFκB p65 subunit, PI3K = phosphatidylinositol-4,5-bisphosphate 3-kinases, Rac1 = Rac family small GTPase 1, RIPK2 = receptor interacting serine/threonine kinase 2, SOCS1 = suppressor of cytokine signaling 1, STAT = signal transducer and activator of transcription, TAB = TGF-beta activated kinase 1 binding protein, TAK1 = TGF-beta activated kinase 1, TLR = toll-like receptor, TNF-α = tumor necrosis factor-alpha, TRAF6 = TNF receptor associated factor 6, TRAM = TIR domain containing adaptor molecule 2, TRIF = TIR domain containing adaptor molecule 1, and TYK2 = tyrosine kinase 2.

Based on the biochemical composition of their cell wall, pathogens mediate a complex set of signals [32,33]. Stimulation of multiple TLR types is associated with more sustained signaling events than stimulation of single TLRs in order to elicit an adapted macrophage response in terms of type and amplitude [18,33]. Indeed, the described M1 and M2 subsets represent two extremes in a continuum of activation states [5,7,18,31]. Macrophage polarization is a dynamic and reversible process that is controlled by a variety of signaling cascades and feedback mechanisms [3,6,7,8,12,18,31]. Activation of the TLR signal pathway by either TLR4 or TLR2 stimulation is accompanied by the initiation of negative feedback loops for return to the physiological state after successful pathogen control (resolution; Figure 2) [3,18,21,34,35]. This involves the adjustment of mRNA and protein levels of relevant mediator and inhibitor proteins by either promotion or blockade of transcription, mRNA stability, and translation [24].

As a result of the MyD88-dependent signaling pathway induced by both TLR4 and TLR2, activation of the protein kinase p38 leads to phosphorylation of the transcription factor CREB and recruitment of coactivators of the p300-CBP family [36,37], which ultimately leads to the activation of the transcription factors KLF4 and C/EBPβ [31,38]. Both KLF4 and C/EBPβ have been described to mediate M2 polarization [2,4,18,31]. KLF4 induces the M2 marker protein arginase 1 [2,3]. In addition, KLF4 promotes the expression of another transcription factor that is associated with M2 polarization: PPAR-γ [3,4,18]. Accordingly, KLF4 activation is associated with downregulating TNF-α and key oxidative burst enzymes (iNOS, COX-2) [2,3]. C/EBPβ, in turn, competes with NFκB for promoter binding sites and mediates an increase in the expression of arginase 1 and IL-10 [2,31,39]. IL-10 is an anti-inflammatory messenger that acts synergistically with IRF3-induced interferon-beta (IFN-β) via the JAK-STAT pathway to support the activation of STAT3 and STAT6 [3,13,18,37]. STAT3 and STAT6 inhibit p38, ERK, and JNK kinases and are well-described mediators of M2 polarization [2,3,4,7,18]. Through the Akt1 pathway, IL-10 also promotes the synthesis of the inhibitor protein IRAK-M, which inhibits the signaling cascades of TLR4 and TLR2 by targeting MyD88 and IRAK4 [12,24,34,40].

Another feedback mechanism is the TLR-induced upregulation of SOCS1. LPS binding to the TLR4 receptor leads to an increase in SOCS1 expression within 2 h. In addition, the PI3K-Akt pathway, which is particularly activated after TLR2 stimulation, has been described to play a special role in TLR-mediated upregulation of SOCS1 [3,18,25,30,34,41,42]. SOCS1 also mediates the blockade of IRAK4 [41]. In addition, a SOCS1-dependent inhibition of IRAK1 and of the NFκB family member p65 has been described [5,30,41]. 

Furthermore, activation of NFAT family transcription factors has been described after stimulation of the TLR cascade, particularly after TLR4-mediated LPS stimulation [43]. By interacting with AP-1, this leads to transcriptional suppression of the cytokines IL-1β, IL-6, and TNF-α [43]. Thus, controlled by the TLR cascade and coactivated pathways, a network of overlapping and temporally redundant regulatory mechanisms exists that is fundamental for fine-tuning macrophage polarization.

## 3. Impact of Post-Transcriptional Regulatory Mechanisms

### 3.1. miRNAs

Macrophage polarization can be influenced at both the transcriptional and post-transcriptional levels. Transcription involves translating genetic information stored in DNA into a transcript, called mRNA, which is the starting point for the synthesis of gene products (i.e., proteins). The signaling cascades induced after the detection of bacterial stimuli lead to the activation of specific transcription factors that regulate the rate of synthesis and, thus, the amount of mRNA available in the cell by interacting with specialized promoter regions, enhancers, or silencers. Post-transcriptional regulation is to be distinguished from the transcriptional control. Here, gene expression is influenced by interacting with the already transcribed mRNA, whereby both mRNA stability and translational efficiency can be altered [44]. Post-transcriptional regulation was first described in 1993 in the context of small noncoding RNAs and micro-ribonucleic acids (miRNAs) [45] and continues to be studied.

miRNAs are a distinct group of single-stranded ribonucleic acids of 19 to 24 nucleotides in length that act primarily as post-transcriptional regulators of gene expression [46,47]. The genes that encode miRNAs are scattered throughout the genome. The majority of them have been assigned their own promoters [48]. However, about a quarter of human miRNA genes have been shown to be located in intron regions of protein-coding genes and are under the control of the promoters of those protein-coding genes [49]. Some miRNA genes are localized within the DNA and are in close proximity to each other. These miRNA gene clusters are under the control of a joint promoter [50]. MiRNAs of a cluster often belong to a miRNA family, which is characterized by the similarity of the nucleotide sequences of the individual family members. The extent to which the clusters also bundle miRNAs with functional similarities has yet to be conclusively clarified [51].

Transcription of miRNA genes in the nucleus is mainly mediated by RNA polymerase II, resulting in the formation of a hairpin primary miRNA (pri-miRNA) of approximately 80 nucleotides in length. Post-transcriptional modification of the pri-miRNA involves the synthesis of a 7-methylguanylate at the 5′-end and a polyadenylate chain at the 3′-end [52]. The modified pri-miRNA is truncated by approximately 70 nucleotides by a nuclear enzyme complex consisting of the RNA-binding protein Pasha and the RNase III Drosha to form the precursor miRNA (pre-miRNA) and is then actively transported out of the nucleus by exportin-5 [53]. In the cytoplasm, the hairpin structure is removed by the enzyme complex Dicer, resulting in a double-stranded miRNA duplex of approximately 22 nucleotides in length [50]. Through interaction with the RNA-induced silencing complex (RISC), this miRNA duplex is unwound to form two complementary single-stranded mature miRNAs [54].

The interaction of a mature miRNA with RISC leads to the formation of miRNA-containing RISC (miRISC), which in turn affects the translation of so-called target mRNAs. Changes in a cell’s miRNA expression profile thus serve to fine-tune cellular protein expression and cell functionality. Both the binding site of the miRNA on the target mRNA and the degree of complementarity of this base pairing determine the effect on mRNA expression. A high complementarity of base pairing within the seed region of the miRNA with the 3′-end of the target mRNA is reportedly associated with a reduced translation as well as the decapping and deadenylation of this mRNA [51]. Interactions between miRNA and the 5′-end or coding sequences of a target mRNA, however, were associated with gene silencing [55,56]. An increase in the expression of a miRNA, therefore, is assumed to enforce post-transcriptional inhibition of its target genes, whereas a decrease in miRNA expression levels is usually accompanied by a promotion in the translational efficiency of target genes [46,47,57,58]. However, the interaction between miRNA and mRNA can also have a positive effect on gene expression [59]. MiRNA-based regulation is estimated to affect approximately 60% of human protein-coding genes [60]. Indeed, one miRNA may recognize and affect different mRNAs, and one mRNA may be the target of different miRNAs. The complexity of miRNA-based post-transcriptional regulation is further increased by the fact that miRNAs are expressed in a cell- and tissue-specific manner [48].

Since the discovery of the first miRNA “lin-4” in a nematode by Lee et al. in 1993 [45], new miRNAs have also been identified in humans. The miRBase database, established in 2002, collects information on miRNA sequences, nomenclature, and target genes [61,62,63]. The current release 22.1 lists a total of 38.589 precursor miRNAs and 48.860 mature miRNAs from 271 different organisms. The significant increase in the number of miRNAs identified in recent years is a reflection of both the importance of post-transcriptional regulation and the consequent interest of the research community.

### 3.2. miRNA Expression Profiles of Macrophages from the M1 versus the M2 Activation Spectrum

Studies have reported associations between altered miRNA expression levels and various pathological conditions, including inflammatory processes [25,57]. Accordingly, several groups have investigated to what extent the miRNA expression profile changes following macrophage polarization. Table 2 summarizes relevant miRNAs that have been documented to be altered compared to unstimulated macrophages. To date, it is generally agreed that polarization of macrophages is associated with altered miRNA expression [4,7,28,58,64,65]. There is also consensus that there are differences in the miRNA profile as a function of the stimulation scenario and the corresponding polarization to the M1 or M2 end of the activation spectrum [4,7,28,58,64,65]. It should be noted, however, that the miRNAs identified as having altered expression levels vary from one study to another (Table 2). Only an increased expression of miR-155-5p in M1 polarization is consistently reported (Table 2; [11,58,66,67,68,69,70,71,72,73]). The interstudy variability, in spite of the analysis of polarized macrophages of the same phenotype, points to the existence of additional influencing factors. The existing studies differ with respect to relevant points such as the time between stimulation and expression analysis as well as macrophage origin (e.g., species differences; primary vs. immortalized cells), limiting comparability. Those methodological differences between the studies suggest that both the timing and the cell origin may be relevant for the miRNA expression profile.

It should be noted that the consequences of TLR4 stimulation on the miRNA profile of macrophages have so far been the focus of research, whereas the consequences of TLR2 stimulation are almost unknown. Thus, there are virtually no studies comparing the effects of TLR4 stimulation with those of TLR2 stimulation. As a result, there is a risk of bias in the evidence base. The lack of data is worrisome as comparative studies of macrophages stimulated by Gram-positive (i.e., LTA, *R. equi*) or Gram-negative (i.e., LPS, *P. aeruginosa*) agents support the hypothesis that the miRNA expression profile of M1 macrophages may vary, at least in part, depending on the activation of the TLR4 and TLR2 cascades, respectively [66]. The miRNAs associated with TLR2 activation in our study include miR-7a-5p, miR-148a-3p, miR-155-5p, and miR-351-5p [66]. An increase in miR-155-5p expression after TLR2 stimulation has also been reported in other studies [73].

### 3.3. TLR Signaling Cascade and miRNA Expression

miRNAs have been implicated as critical post-transcriptional regulators of macrophage polarization [4,7,28,58,64,65]. Simultaneously, miRNA expression is subject to the influence of the TLR signaling cascade [7,25,58,65]. Besides transcriptional induction or repression, an influence on the stability of miRNA precursors formed during transcription has been described. Indeed, as early as 2007, O’Connell et al. speculated that the altered expression of miR-155-5p after TLR2 stimulation is mediated by AP-1 since binding sites for this transcription factor are found in the promoter of the miR-155 gene [11,25,83]. It is interesting to note that most miRNA genes that are associated with M1 polarization have binding sites for TLR-induced transcription factors. These include subunits of AP-1 such as c-Fos, Fosl2, c-Jun, JunB, and JunD [84], transcription factors cooperating with AP-1 such as ATF4, NFATc1, and NFATc3 [43,85,86], the transcription factors Bcl6 and Bcl6B, which mediate the suppression of AP-1 activity [87], as well as the transcription factors Max and Myc, whose expression is under the influence of AP-1 [66,88]. The mutual interaction of the TLR signaling cascade and miRNA expression results in a complex regulatory process based on positive and negative feedback loops.

The observation of a hierarchy of miRNA expression adds another level of complexity [57,89]. Evidence suggests a temporally differentiated synthesis of specific miRNAs after TLR induction [89,90]. Changes in the expression levels of the so-called early miRNAs occur immediately after ligand binding. For example, miR-155-5p was reported to be upregulated within 2 h of stimulation [57,72,91,92,93]. As a higher-ranking miRNA, miR-155-5p then regulates the expression of further miRNAs of the late response [57,74,89]. The resulting differences in macrophage miRNA expression profiles implicate post-transcriptional miRNA-mediated regulation in both the induction of M1 polarization immediately after TLR signaling initiation and in delayed negative feedback loops [90].

### 3.4. Macrophage Polarization Influenced by miRNAs

**Figure 3:**
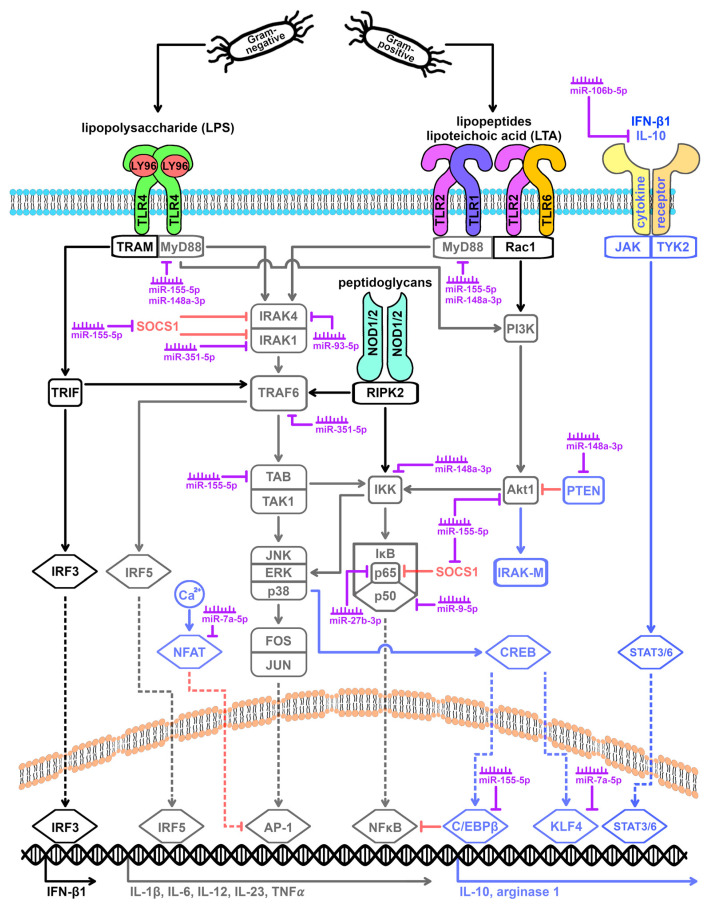
miRNA targets within the TLR4 and the TLR2 signaling cascade. Activation of TLR4 by Gram-negative bacteria and of TLR2 by Gram-positive bacteria leads to changes in the miRNA expression profile of macrophages. Various mediators of the TLR signaling cascade are target genes of differentially expressed miRNAs, forming a feedback mechanism. To what extent the stimulating agent (Gram-negative versus Gram-positive) and the activated TLR family member (TLR4 versus TLR2) influence the type and/or direction of expression of altered miRNAs is unclear from the current literature. However, it is well established that miR-155-5p plays a distinct role in the post-transcriptional fine-tuning of macrophage polarization and is upregulated after both TLR4 and TLR2 stimulation. AP-1 = activating protein-1, Akt1 = AKT serine-threonine protein kinase 1/protein kinase B, C/EBPβ = CCAAT enhancer binding protein beta, CREB = cAMP responsive element binding protein, ERK = extracellular signal-regulated kinases, FOS = Fos proto-oncogene, IFN-β1 = interferon beta 1, IκB = NFκB inhibitor, IKK = IκB kinase, IL = interleukin, IRAK = interleukin 1 receptor associated kinase, IRF = interferon regulatory factor, JAK = Janus kinase, JNK = c-Jun N-terminal kinase 1, JUN = Jun proto-oncogene, KLF4 = KLF transcription factor 4, LY96 = lymphocyte antigen 96, MyD88 = myeloid differentiation primary response 88, NFAT = nuclear factor of activated T cells, NFκB = nuclear factor of kappa light polypeptide gene enhancer in B-cells, NOD = nucleotide binding oligomerization domain, p38 = mitogen-activated protein kinase 14, p50 = NFκB p50 subunit, p65 = NFκB p65 subunit, PI3K = phosphatidylinositol-4,5-bisphosphate 3-kinases, PTEN = phosphatase and tensin homolog, Rac1 = Rac family small GTPase 1, RIPK2 = receptor interacting serine/threonine kinase 2, SOCS1 = suppressor of cytokine signaling 1, STAT = signal transducer and activator of transcription, TAB = TGF-beta activated kinase 1 binding protein, TAK1 = TGF-beta activated kinase 1, TLR = toll-like receptor, TNF-α = tumor necrosis factor-alpha, TRAF6 = TNF receptor associated factor 6, TRAM = TIR domain containing adaptor molecule 2, TRIF = TIR domain containing adaptor molecule 1, and TYK2 = tyrosine kinase 2.

miRNAs are discussed as key regulators of the TLR signaling cascade, which influence the expression of the TLR receptors themselves, as well as TLR adaptor and cascade molecules, TLR-activated transcription factors, TLR-activated cytokines, and TLR cascade repressors (Figure 3) [22,25,57,58,90]. Although the available evidence is primarily based on studies of the TLR4 signaling cascade, current knowledge suggests that macrophage polarization leads to a change in the miRNA expression profile regardless of the type of stimulating agent (Gram-negative versus Gram-positive) and the TLR signaling cascade induced by it (TLR4 versus TLR2). Furthermore, the available evidence indicates that both the miRNAs altered after TLR4 stimulation and those altered after TLR2 stimulation have a feedback effect on the signaling cascades induced by these receptors. Intriguingly, miRNAs mediate both pro- and anti-inflammatory effects in the immune system [4,7]. It is described that miRNAs act in a temporally staggered manner on different target genes to counterbalance excessive immune responses (i.e., massive defense reactions as well as exaggerated immune suppression) [90]. Besides the temporal orchestration, the preferential influence on a specific target gene apparently depends on its expression level [25]. Accordingly, miRNA may play an important role in the switch between the early inflammatory response and the resolution phase [11,57].

miRNAs can affect the cellular levels of target proteins by both mRNA destabilization and translational repression [23,46]. A pure translational repression, i.e., a reduction in protein levels at constant mRNA levels, as it has been described for MyD88 and TAB2 by miR-155-5p [25,47], is currently estimated to be limited to 6–26% of cases [46]. Thus, changes in TLR mediator mRNA expression levels after macrophage stimulation may be both transcriptional and post-transcriptional. In addition to influencing mRNA and protein expression, accelerated protein degradation and modulation of a protein’s activity status are relevant targets that can be both miRNA-mediated and complementary to miRNA-based regulation [57]. Examples of the interplay of different regulatory mechanisms are the mediator proteins TRAF6 and Akt1, as well as the NFAT transcription factor family.

TRAF6 plays a central role in both MyD88-dependent and MyD88-independent signaling. It has been described that TRAF6 is ubiquitinated and proteosomally degraded in an IRAK1-dependent manner in the context of negative feedback [28]. In addition, our own studies show a downregulation of TRAF6 mRNA levels after TLR4 or TLR2 stimulation, which is accompanied by an upregulation of the miRNA miR-351-5p [66,94]. TRAF6 has been described as a target gene of miR-351-5p [95]. Interestingly, miR-351-5p also targets IRAK1 [95]. Thus, miR-351-5p may inhibit TRAF6 but may also counteract IRAK1-mediated degradation of TRAF6, thereby helping to maintain a critical balance.

The mediator protein Akt1 promotes M1 polarization via activation of NFκB [8]. However, it is also part of the IL-10-mediated negative feedback [12,13,37,96]. We observed that Akt1 mRNA levels are downregulated following TLR4 or TLR2 stimulation [94]. This is in line with the description of Akt1 as a target gene of the miRNA miR-155-5p [66,97], whose expression has been consistently reported to be upregulated by various members of the TLR family [4,5,7,11,12,20,24,25,34,42,47,58,70,71,72,73,83,90,91,92,98,99,100,101,102,103]. In addition, Akt1 activity is subject to post-transcriptional modulation. Akt1 is negatively regulated by the phosphatase PTEN, which in turn can be inhibited by the miRNA miR-148a-3p [79]. MiR-148a-3p is upregulated both after TLR4 and TLR2 stimulation [66,79,80], resulting in miRNA-mediated promotion of Akt1. Thus, a post-transcriptional balancing of expression levels and activity status ensures a fine-tuning of pro- and anti-inflammatory effects.

Changes in activity status after TLR stimulation are also discussed for NFAT family transcription factors as part of TLR-induced feedback loops [43]. On the other hand, the mRNA levels of NFATC1 are downregulated [94], counteracting the negative feedback. A possible explanation for this is the TLR2-induced upregulation of the miRNA miR-7a-5p, which targets NFATC1 and NFATC3 [66,97]. Again, this seems to be another instance of post-transcriptional refinement.

Further TLR-associated target genes of miRNAs include the mediator proteins MyD88, IRAK4, and IKK-beta, the transcription factors NFκB, KLF4, and PPAR-γ, and the cytokine IL-10. IRAK4 is targeted by the miRNA miR-93-5p, which has been consistently reported to be downregulated following LPS stimulation, thereby promoting M1 polarization [75,76,104,105]. M1 polarization is also associated with upregulation of the miRNA miR-7a-5p induced after TLR2 stimulation, which, in addition to NFAT, also targets the M2-associated transcription factor KLF4 [106]. There is also evidence that miR-7a-5p affects the TLR4 signaling cascade. In a mouse model of LPS-induced acute lung injury (ALI), increased KLF4 levels and reduced pathology have been described as a consequence of miR-7 deficiency [106]. In contrast, TLR-induced upregulation of miR-148a-3p is primarily associated with M2 polarization [79,107], as this miRNA inhibits translation of the mediator protein MyD88 [80] and blocks release of the transcription factor NFκB by negatively affecting IKK-beta [108]. Another critical role in M1-M2 polarization is played by miR-106b-5p, which post-transcriptionally inhibits the M2 inducer IL-10 [57,90]. Interestingly, the expression of miR-106b-5p is decreased after LPS stimulation [77]. Thus, M1 macrophages have lower levels of miR-106b-5p compared to M2-type macrophages [77]. This counteracts a one-sided polarization of the macrophages. The seemingly contradictory effects of the miRNAs miR-9-5p and miR-27b-3p can probably be interpreted in a similar way. For both miRNAs, overexpression is linked to inhibition of both the proinflammatory transcription factor NFκB [5,21,25,57,76,90,92,109,110,111,112] and the anti-inflammatory transcription factor PPAR-γ [11,28,57,74,90,92].

The differential effect of miR-155-5p is another prominent example of the yin-yang effect of miRNAs in macrophage polarization. MiR-155-5p is upregulated following TLR stimulation mediated by the transcription factors AP-1 and NFκB [83,90,98,99,113] and is considered critical for the polarization of macrophages to the M1 phenotype [4,11,18,20,58,91]. Indeed, knocking out miR-155-5p in macrophages is associated with reduced clearance of *Streptococcus pneumoniae* or *Mycobacterium bovis* [70,71]. The proinflammatory effect of miR-155-5p is based on post-transcriptional blockade of inhibitor proteins of the TLR signaling cascade, such as SOCS1, as well as mediators of M2 polarization, such as C/EBPβ [4,5,7,8,11,18,20,25,30,34,39,41,42,47,57,58,71,72,89,90,92,93,98,102,103,114,115]. Furthermore, miR-155-5p mediates the stabilization of TNF-α mRNA [4,5,7,34,57,65,90,102,113]. Besides these proinflammatory effects, miR-155-5p also acts as an anti-inflammatory effector. Among others, miR-155-5p causes the translational repression of the TLR cascade proteins MyD88 and TAB2 and mediates the mRNA degradation of IKK-epsilon [4,5,7,20,24,25,35,47,57,72,76,90,91,92,93,100,101,102,113,114,116]. Furthermore, Akt1 and PKI-alpha, an inhibitor of protein kinase A, are targeted by miR-155-5p [71]. Inhibition of PKI-alpha is associated with the promotion of the cAMP/CREB pathway and, thus, M2 polarization [38]. It is noteworthy that there is also an IL-10-mediated feedback. As described, stimulation of the TLR signaling cascade causes upregulation of IL-10 [96], which in turn inhibits the expression of miR-155-5p [4,20,57,65,90,98,116], thereby promoting SOCS1 expression [4,8,47,90].

In conclusion, the present findings implicate miRNAs in macrophage homeostasis mechanisms. Appropriate macrophage polarization is highly important for human health. This fits with the current discussion of miRNAs as fundamental factors in a wide variety of pathogenesis, such as inadequate defense against pathogens, chronic inflammatory reactions, and oncogenic diseases [25,57].

## 4. Final Remarks

The post-transcriptional regulation of TLR-induced M1 polarization is not well understood. Few studies have examined the effects of Gram-negative or Gram-positive stimulation of macrophages on the miRNA profile and the resulting influence on immune cell polarization. This is complicated by significant variances in the miRNAs being discussed. For PCR-based miRNA quantification, the use of appropriate reference genes is crucial. Many reported reference genes, however, show altered expression under inflammatory conditions [117]. Therefore, it is necessary to experimentally demonstrate that the selected reference gene is stably expressed and independent of external conditions. Digital PCR may be used to quantify miRNAs accurately [118]. The question of medical relevance can only be answered by establishing valid evidence based on macrophage miRNA expression profile after TLR stimulation, the target genes affected, and the potency of induced adjustments for M1 polarization.

The immunological importance of miRNAs is clearly demonstrated by studies in miRNA knockout (KO) mouse models. MiR-146a-deficient mice develop a spontaneous autoimmune-like disease that leads to death within 6 months after birth [65]. The KO mice exhibit immune tolerance and macrophage hyperreactivity to LPS [65]. In addition, the mice develop tumors in secondary lymphoid organs, which is likely due to chronic inflammation [65]. It has been reported that miR-146a-deficient mice exhibit exacerbated inflammation, with infiltrating cells accumulating in the dermis and increased cutaneous inflammation [23]. In addition, aberrant expression of miR-146a is associated with a dysregulation of the innate immune response and a chronic dysregulation of the NF-kB signaling pathway, resulting in a phenotype with features of a myeloid malignancy [23]. MiR-155 KO mice have been shown to have a compromised immune response to *Salmonella typhimurium* and are unable to be successfully vaccinated against the pathogen [65]. The mice retain the ability to prevent acute invasive pneumococcal *Streptococcus pneumoniae* infection but have significantly higher bacterial loads after colonization [70]. This has been attributed to a reduced ability to generate high-titer *Streptococcus pneumoniae*-specific antibodies and a reduced ability to induce Th17 cell polarization [70]. Further analysis revealed a defect in the activation of B cells and T cells, which explains the lack of immunogenicity in these mice [65,116]. The failure of the T cell response was due in part to the inability of DCs to present antigen and an altered Th1 response in which CD4^+^ T cells had impaired cytokine production [65]. Another study showed that miR-155 KO mice had decreased numbers of germinal center B cells, while miR-155 overexpression mice had increased levels [65]. MiR-155-deficient mice are characterized by Ig class switching (likely due to aberrant activation-induced cytidine diamine (AID) expression), lower IgM levels, reduced IL-2 and IFN production, and Th2 differentiation tendency [101]. In miR-7 knockdown mice generated by miRNA sponge technology, it was found that miR-7 deficiency ameliorated the pathologies of LPS-induced acute lung injury (ALI) [106]. MiR-7 deficiency resulted in reduced levels of bronchoalveolar lavage (BAL) and proinflammatory cytokines. MiR-7 deficiency also significantly altered the proportion and number of various immune cells in BAL [106].

Dysregulation of miRNA expression has been implicated in the pathogenesis of immunologic human diseases, suggesting that miRNAs may serve as novel diagnostic or therapeutic targets. In a mouse model of LPS-induced ALI, miR-351-5p antagomir ameliorated LPS-induced oxidative stress and inflammation in the lung, whereas miR-351-5p agomir exacerbated LPS-induced oxidative stress and inflammation [119]. Similarly, using an in vivo mouse model of LPS-induced ALI, agomiR-93 was able to attenuate lung injury as evidenced by decreased lung permeability, reduced lung wet/dry weight ratio, and increased survival of the mice [104]. At the same time, agomiR-93 significantly reduced LPS-induced interleukin IL-6, IL-1ß, and TNF-α levels in BAL [104]. Experiments in an ovalbumin-induced model of allergic asthma showed that suppression of miR-146a expression attenuated allergic asthma by increasing Th1 cytokines [22]. Thus, it may be possible to regulate inflammatory processes by manipulating key miRNAs. Knowing how to modify the microtranscriptome could, therefore, be useful in developing new therapies targeting miRNAs, which in turn could restore or maintain immune system homeostasis altered by an excessive inflammatory response [76]. Since miRNAs can regulate multiple proteins, additional studies are urgently needed to elucidate their therapeutic potential [76]. MiRNA targeting for therapeutic purposes is still in its infancy. However, they remain of interest for future drug development, given the role of certain miRNAs in the control of inflammation and, in particular, macrophage function.

## Figures and Tables

**Table 1:** Overview of known mammalian TLRs and their ligands [15].

TLR	Ligands
TLR1	Triacyl lipopeptides
TLR2	Peptidoglycan, lipopeptides, LTA, lipoarabinomannan, GPI anchors, phenol-soluble modulin, zymosan, glycolipids
TLR3	Double-stranded RNA
TLR4	LPS, Taxol, RSV fusion protein, MMTV envelope protein
TLR5	Flagellin
TLR6	Diacyl lipopeptides
TLR7	Single-stranded RNA, imidazoquinolines
TLR8	Single-stranded RNA, imidazoquinolines
TLR9	CpG DNA
TLR10	Unknown
TLR11	Profilin, flagellin
TLR12	Profilin
TLR13	Bacterial 23S risbosomal RNA

**Table 2:** Overview of miRNAs with alterations in expression after macrophage polarization.

miRNA	Associated Activation Spectrum	Direction of Expression Change	Reference(s)
miR-7a-5p	M1	upregulation	[66]
miR-9-5p	M1	downregulation	[66]
		upregulation	[11,21,58,67]
miR-27b-3p	M1	downregulation	[66]
		upregulation	[74]
miR-29b-3p	M1	upregulation	[68]
miR-93-5p	M1	downregulation	[66,75,76]
miR-106b-5p	M1	downregulation	[66,77]
miR-127-3p	M1	upregulation	[11,78]
miR-143-3p	M1	downregulation	[11,69]
miR-145-5p	M1	downregulation	[11,69]
miR-147-3p	M1	upregulation	[67]
miR-148a-3p	M1	upregulation	[66,79,80]
miR-155-3p	M1	upregulation	[11,67]
miR-155-5p	M1	upregulation	[11,58,66,67,68,69,70,71,72,73]
miR-204-5p	M1	upregulation	[11,69]
miR-351-5p	M1	upregulation	[66]
miR-451	M1	upregulation	[11,69]
let-7c	M2	upregulation	[67]
miR-23a-5p	M2	downregulation	[11]
		upregulation	[67]
miR-27a-3p	M2	upregulation	[67]
miR-34a	M2	downregulation	[11,81]
miR-99b	M2	downregulation	[68]
miR-124	M2	downregulation	[11]
miR-132	M2	upregulation	[11,82]
miR-193b-3p	M2	upregulation	[11,68]
miR-200a-3p	M2	upregulation	[11]
miR-223	M2	downregulation	[11]
miR-511	M2	upregulation	[68]

## Data Availability

Not applicable.
